# Round the Hospitals

**Published:** 1916-11-11

**Authors:** 


### ROUND THE HOSPITALS.

In the House of Commons last week the question of the State Registration of Nurses was referred to. The Prime Minister, asked by Major Chapple whether he would bring in a Bill on similar lines to the one submitted by the Central Committee for the State Registration of Nurses, stated that he could not at the present time undertake to introduce it. As none but Government Bills are likely to be heard during war-time the Bill of the Central Registration Committee is, therefore, indefinitely shelved. Meanwhile the College of Nursing is proceeding with its Register and its own Registration Bill. The College Bill provides for a democratic Council, Clause 4 (*a*) enacting that not less than two-thirds of the Council shall be elected by the nurses on the General Register. It is further provided in the College Bill that every nurse registered under the Act shall without further fee become a member of the College of Nursing. The fee for registration is 21s. In all previous Registration Bills which have been presented to Parliament the fees have been much higher, and under the latest Bill of the Central Committee, i.e., that for 1914, the fees for examination and registration may be as much as five guineas, with the addition of a subscription of 2s. 6d. a year, in default of which payment the nurse's name is to be removed from the Register. Matrons, sisters, and nurses should address themselves earnestly to the question of registration, and should write to Miss Rundle, the Secretary of the College of Nursing, at 6 Vere Street, Cavendish Square, on any point which is not clear to them.

We deeply regret to learn that Miss L. V. Haughton, matron of Guy's Hospital, was taken ill on Thursday of last week with an attack of cerebral hæmorrhage, and is still in a very serious condition. Her self-sacrificing devotion to her work and keen interest in the welfare of her nurses have endeared her to a large circle of friends, whose sympathy goes out to her in her grave illness. Letters of regret and inquiry have been received from Her Majesty Queen Alexandra, from the Countess of Airlie, and many others. Trained at Guy's Hospital, Miss Haughton was matron at Sir Patrick Dun's Hospital, Dublin, from 1902 to 1909, when she returned to her old training-school as matron. Miss Haughton has not been in her usual health for some time past, but has carried on her work, and even increased it owing to the interest she has displayed in the foundation of the College of Nursing and her ardent desire to see it firmly established, and State Registration under an agreed Bill made an accomplished fact.

Great improvements have been effected during the last three years in the organisation of the teaching of the nurses at St. Thomas's Hospital, the strengthening of the examinations, and the practical tests which are applied. The results of these developments were seen last week when the first distribution was made of the Nightingale Medal. This is to be awarded every year, in gold, silver, and bronze, to the three students whose work shall seem to mark them as pre-eminent in their year. To qualify for a gold medal at least 75 per cent. of the total number of marks must be obtained, the marks including those for conduct and general efficiency. The Nightingale Medal has been founded by an old Nightingale nurse, and designed by Countess Feodora Gleichen, whose skill has wrought into it many memories of the foundress of the Nightingale School. It is a beautiful production and cannot fail to be a much-coveted distinction, and the proud possession of those nurses who may become qualified to receive it.

The vacancy in the matronship of Ancoats Hospital, Manchester, has been filled by the appointment of Miss Maud Earl, who since 1911 has been matron of the Princess Alice Hospital, Eastbourne. Miss Earl was trained at St. Bartholomew's Hospital, and was in charge of the operating theatres there for 6½ years. The late matron of Ancoats Hospital, Miss Lucy Beard, has recently retired on her marriage.

The suggestion has been put forward that any difficulty there may be in finding nursing assistants or V.A.D. helpers for the military hospitals would be entirely solved if each matron recruited her own, as in a civil hospital. It is held that the existing system of getting them is full of red-tape, very cumbersome, and often results in a worse selection than if the matter were left to the matron. This is a point which might well be considered. In the case of new hospitals the present plan might be continued, but in all hospitals which have been established for a year it is urged that the matron should have no difficulty in recruiting the assistants herself.

One whole day off duty in each seven is a privilege which many matrons would like to be able to grant to every member of their nursing staff, but it is one which would involve a considerable addition to its number. At the West Ham Union Infirmary, with a nursing staff of 125, extra nurses to the number of twenty-two would be required to enable a weekly day off duty to be arranged. The matron and medical superintendent have been anxious for this reform, and the Guardians, to their great credit, were willing to authorise the necessary staff additions to enable it to be brought about, but up to the present, we understand, the proposal has been negatived by the Local Government Board on the ground that other Unions opposed it.

An interesting meeting, at which Princess Henry of Battenberg was present, was held on November 3, under the auspices of the British Women's Emigration Association and the Colonial Nursing Association, at Sunderland House, Mayfair, to consider the great need for midwives and maternity nurses in Canada. Lady Piggott, who was in the chair, stated that the midwife trained solely for that work is not recognised in many parts of Canada, and the fully trained hospital nurse usually exhibits a strange repugnance to that branch of nursing, or to leaving the cities for the newly settled districts. It is desired to select suitable women, whose homes have been broken up by the war, for training in midwifery, and to settle them in centres from which they can go and nurse in the surrounding country. The scheme has the cordial approval of the various women's organisations of Canada. An important point, which was emphasised by Miss Amy Hughes, is to secure the right women, who must be strong mentally, morally, and physically, with initiative and common sense in addition. It is suggested that if a system of nursing can be established in connection with the municipal hospitals, it will guarantee to the mothers the help they require. The Canadian Sub-Committee of the Colonial Nursing Association has been formed, with Lady Piggott as chairman, to further some such scheme and to co-operate with the Dominion in its endeavour to place adequate midwifery within the means and reach of pioneer settlers in outlying districts.

A matron sends us the following recipe for a good floor polish. Mix in a double receptacle, over a fire, 1 gallon of turpentine, 6 oz. of paraffin, 4 oz. of bees'-wax. Add 1 oz. of liquid ammonia to each pint after the mixture has cooled. She also tells us that she finds Indian, and especially Mirzapore, rugs can be successfully washed in the hospital laundry. They are put in the machines with a good soap solution to which ammonia has been added, and having been rinsed in several waters, are placed, without wringing or any attempt at squeezing out the water, on the drying horses. When dry, a good brushing with a hard brush will make the rug look like new.

**For Matrons' and Sisters' Appointments, see p. 128.**

